# Identification and validation of suitable endogenous reference genes for gene expression studies in human peripheral blood

**DOI:** 10.1186/1755-8794-2-49

**Published:** 2009-08-05

**Authors:** Boryana S Stamova, Michelle Apperson, Wynn L Walker, Yingfang Tian, Huichun Xu, Peter Adamczy, Xinhua Zhan, Da-Zhi Liu, Bradley P Ander, Isaac H Liao, Jeffrey P Gregg, Renee J Turner, Glen Jickling, Lisa Lit, Frank R Sharp

**Affiliations:** 1 Department of Neurology and M.I.N.D. Institute, University of California at Davis Medical Center, Sacramento, CA 95817, USA; 2 Department of Pathology, and M.I.N.D. Institute, University of California at Davis Medical Center, Sacramento, CA 95817, USA

## Abstract

**Background:**

Gene expression studies require appropriate normalization methods. One such method uses stably expressed reference genes. Since suitable reference genes appear to be unique for each tissue, we have identified an optimal set of the most stably expressed genes in human blood that can be used for normalization.

**Methods:**

Whole-genome Affymetrix Human 2.0 Plus arrays were examined from 526 samples of males and females ages 2 to 78, including control subjects and patients with Tourette syndrome, stroke, migraine, muscular dystrophy, and autism. The top 100 most stably expressed genes with a broad range of expression levels were identified. To validate the best candidate genes, we performed quantitative RT-PCR on a subset of 10 genes (TRAP1, DECR1, FPGS, FARP1, MAPRE2, PEX16, GINS2, CRY2, CSNK1G2 and A4GALT), 4 commonly employed reference genes (GAPDH, ACTB, B2M and HMBS) and PPIB, previously reported to be stably expressed in blood. Expression stability and ranking analysis were performed using GeNorm and NormFinder algorithms.

**Results:**

Reference genes were ranked based on their expression stability and the minimum number of genes needed for nomalization as calculated using GeNorm showed that the fewest, most stably expressed genes needed for acurate normalization in RNA expression studies of human whole blood is a combination of TRAP1, FPGS, DECR1 and PPIB. We confirmed the ranking of the best candidate control genes by using an alternative algorithm (NormFinder).

**Conclusion:**

The reference genes identified in this study are stably expressed in whole blood of humans of both genders with multiple disease conditions and ages 2 to 78. Importantly, they also have different functions within cells and thus should be expressed independently of each other. These genes should be useful as normalization genes for microarray and RT-PCR whole blood studies of human physiology, metabolism and disease.

## Background

Gene expression analysis is widely used to study various biological processes. Different transcript quantification methodologies exist, all of which rely on utilization of proper normalization techniques. Such analysis requires several variables need to be accounted for, including amount and quality of RNA, enzymatic efficiencies, and differences between tissues or cells in overall transcriptional activity. The current, most universally utilized normalization method for PCR and Northern blotting relies on the use of constitutively expressed endogenous reference genes, often termed ''house-keeping genes.'' It has been shown, however, that the expression of such reference genes can vary significantly between different tissues or conditions, thus making it impossible to use the same reference genes for various tissues [1-5]. Moreover, the identification of appropriate control genes presents a circular problem, because in order to find suitable genes for normalization, prior knowledge of their stable gene expression is needed, which also relies on properly normalized data.

The conventional use of a single gene for normalization can introduce a significant error in the quantification of transcript levels [6]. It is recommended that an optimal set of reference genes be identified for each individual experimental setting or tissue. In this study, we used whole-genome human microarrays and surveyed the expression of over 39,500 genes in 526 whole-blood samples from control subjects and patients with Tourette syndrome, stroke, migraine, muscular dystrophy, and autism to identify the best candidate control genes. The 10 best candidate control genes, as well as commonly used house-keeping controls and PPIB, were validated with independent samples of whole-blood RNA from both patients with multiple sclerosis (MS) and age- and gender-matched controls by qRT-PCR. GeNorm was used to identify an optimal set of the most stably expressed genes for normalization in whole-blood expression studies [6].

## Methods

### Patients and samples

We used a large cohort of available human Affymetrx array data that we have generated over the last several years from previous and on-going studies (Table 1). Many, but not all, of the subjects included in our analyses have previously been reported in the following studies: stroke [7-10], Tourette syndrome [11-13], controls [14-18], migraine [19], muscular dystrophy [20,21] and autism [22,23].

**Table 1:** Characteristics of subjects who had gene expression assessed in whole blood on Affymetrix Human microarrarys.

**Diagnosis**	**Patients**			**Controls**		
	**n**	**(M/F)**	**Age**	**n**	**(M/F)**	**Age**
Stroke	108	(76/32)	68	34	(29/5)	51
Tourette	30	(23/7)	14	32	(24/8)	15
Teen Controls	N/A			32	(24/8)	11
Migraine	130	(78/52)	13	10	(6/4)	14
Muscular Dystrophy	51	(43/8)	11	5	(4/1)	11
ASD, Develop Delay	84	(72/12)	4	10	(8/2)	4

The numbers of samples (n), the proportion that came from males versus females (M/F) and the mean age (Age) of the subjects are tabulated for each group. (ASD = Autism Spectrum Disorder)

### RNA Isolation and Quality Control

Whole blood was collected and total RNA was isolated using PAXgene tubes and kits (Qiagen, Valencia, CA, USA). RNA samples were examined for concentration and purity using a Nanodrop ND-1000 spectrophotometer, and integrity was checked using the Agilent 2100 Bioanalyzer. High quality RNA with a RIN number above 8.0 was used for the qRT-PCR experiment. The RIN number takes into consideration not only the conventional ratio of 28 S to 18 S ribosomal RNA, but also additional critical regions of the entire RNA electrophoregram.

### Affymetrix experiments

Affymetrix Human 2.0 Plus arrays (Affymetrix, Santa Clara, CA, USA), surveying over 54,000 probe-sets, were used in this study. The 54,000 probe-sets represent over 39,500 potential human genes (Affymetrix Manual). The standard Affymetrix protocol was followed for the sample labeling, hybridization, and image scanning [23].

### qRT-PCR cDNA synthesis

The cDNA synthesis and real-time PCR was performed at the Lucy Whittier Molecular and Diagnostic Core Facility (University of California, Davis, U.S.A.). The following cDNA synthesis protocol was validated through the Lucy Whittier Molecular and Diagnostic Core Facility using a variety of sample types and species. 10 μl (750 ng) of template RNA per sample, 1 μl gDNA WipeOut Buffer, 1 μl RNase-free water was incubated at 42°C for two minutes and briefly centrifuged. A 1 μL aliquot was TaqMan^® ^analyzed with human GAPDH to confirm all gDNA had been digested. Then 8 μl from the reverse transcription reaction mix (0.5 μl Quantitect Reverse Transcriptase, 2 μl 5× Quantitect RT buffer, 0.5 μl RT Primer Mix, 0.5 μl 20 *pmol *Random Primers, 4.5 μl RNase free water) was added to each well, incubated at 42°C for 40 minutes, inactivated at 95°C for 3 minutes and briefly centrifuged. Finally, 100 μl of water was added to each well and mixed thoroughly.

### Selection of TaqMan Assays

TaqMan assays were selected based on several criteria: 1) only pre-developed and pre-validated gene expression assays were selected from Applied Biosystems (ABI, Foster City, CA, U.S.A.), for which the company guarantees amplification efficiency over 96%. 2) Only _m1 assays (spanning exon -exon boundaries) were selected to ensure no genomic DNA will be amplified. 3) If available, the assays were preferentially selected to amplify the same target sequence where the Affymetrix probe-set was located. 4) If a gene selected for qRT-PCR validation was represented by multiple probe-sets on the Affymetrix array, the standard deviation (SD) of the expression of all probe-sets was inspected and if some were found to have a high standard deviation, their position in the reference sequence was avoided.

### Real-Time TaqMan^® ^PCR

Pre-validated gene expression assays were purchased from Applied Biosystems. See Table 1 for the Assay and Gene IDs. An aliquot of the cDNA from a subset of samples was TaqMan^® ^analyzed with human GAPDH prior to profiling. TaqMan^® ^analysis was done in duplicate reactions using the low density array format as described by Osman et al [24]. This was validated and processed through the Lucy Whittier Molecular and Diagnostic Core Facility (1). The samples were placed in a 384 well plate and amplified in an automated fluorometer (ABI PRISM 7900 HTA FAST, ABI). ABI's standard amplification conditions were used: 2 min at 50°C, 10 minutes at 95°C, 40 cycles of 15 seconds at 95°C and 60 seconds at 60°C. Fluorescent signals were obtained during the annealing temperature and Ct values exported with a threshold of 0.1 and a baseline of 3–10.

### Data Analysis

#### Affymetrix Chips

All 526 arrays were summarized using the GC-RMA algorithm [25]. They were normalized within GeneSpring 7.3 software (Agilent Technologies, Palo Alto, CA) using a three-step normalization procedure, including data transformation, per chip and per gene normalization (GeneSpring Manual).

#### qRT-PCR GeNorm Analysis

All samples for which the standard deviation (SD) of the technical duplicates was ≥ 1.41 (or higher than 2-fold change) were considered missing data points and were not included in the analysis. The GeNorm tool was used to calculate the internal control genes stability measures (M) [6]. In short, input data was generated using the comparative Ct-method [26], where relative expression levels were calculated by setting the lowest Ct value of a particular gene to 1, and the relative expression levels for the same gene in the rest of the samples were calculated. Then for every control gene, the pair-wise variation with all other control genes is estimated in GeNorm as the SD of the log-transformed expression ratios. The internal control gene stability measure M is defined as the average pair-wise variation of a particular control gene with all other control genes. Stepwise exclusion of the gene with the highest M value is performed. Because a pair of control genes is needed to calculate M, the two most stably expressed genes cannot be ranked any further. The optimal number of control genes for normalization is determined by calculating the pair-wise variation V_n_/V_n+1 _between each set of two sequential normalization factors [6]. Consistent with the Vandesompele recommendations, the minimum number of genes for which the V was smaller than 0.15, was used to define the optimal set of control genes for normalization. A normalization factor is calculated based on the geometric mean of the optimal set of control genes.

#### qRT-PCR NormFinder Analysis

The NormFinder Excel plug-in [27] was used as an alternative algorithm to the GeNorm algorithm for ranking the expression stability of the control genes. It ranks the candidate control genes by estimating their expression stability while also taking into account the experimental design (control and disease groups). The input qRT-PCR results were the linearized quantities (2-ΔCt). The main differences between the GeNorm algorithm [6] and the model-based approach in NormFinder [27] is that the latter takes into account the inter- and intra-group variation, as well as the estimation of variances.

#### Analysis of Variance (ANOVA)

Partek Genomics Suite (St. Louis, MI, USA) was used to perform an ANOVA using the Diagnosis as fixed and the Plate as a random factor (μ + Diagnosis + Plate + (Diagnosis × Plate) + ε). The input data were the normalized data to the geometric average of the optimal set of control genes (TRAP1, DECR1, PPIB, and HMBS) using the comparative Ct method.

#### ΔΔCt Method

The **ΔΔ**Ct method [28] was used to calculate the normalized calibrated data. TRAP1, identified by GeNorm as one of the two most stably expressed genes was used as a normalizer. The average of the healthy controls was used as the calibrator. In short, the Ct value of the normalizer was subtracted from the Ct value of each sample (**Δ**Ct). Then the value of the calibrator was subtracted (**ΔΔ**Ct) and the linearized values were calculated (2^-(**ΔΔ**Ct)^). A prerequisite for using this method of analysis is that the normalizer and the gene of interest have very similar amplification efficiency. The ABI assays used in this study have been pre-validated by the company to have over 96% amplification efficiency.

## Results

### Patients and samples

Of the 526 samples, the mean ages of the subjects for each study varied from age 4 (autism study patients and controls) to age 68 (stroke study patients) with a range of 2 to 78 years of age (Table 1). There was a predominance of males (387) to females (139). Control subjects had no known medical, surgical, or psychiatric illnesses. Study subjects had a variety of medical illnesses with varying pathophysiologies, including neurodevelopmental and neurological disorders (autism, mental retardation, Tourette syndrome), stroke (ischemic brain injury), muscular dystrophy (genetic muscle disease), migraine headaches, as well as a presumed autoimmune disease, multiple sclerosis (Table 1).

### Selection of Reference Genes from Whole-Genome Affymetrix Arrays

**Figure 1:**
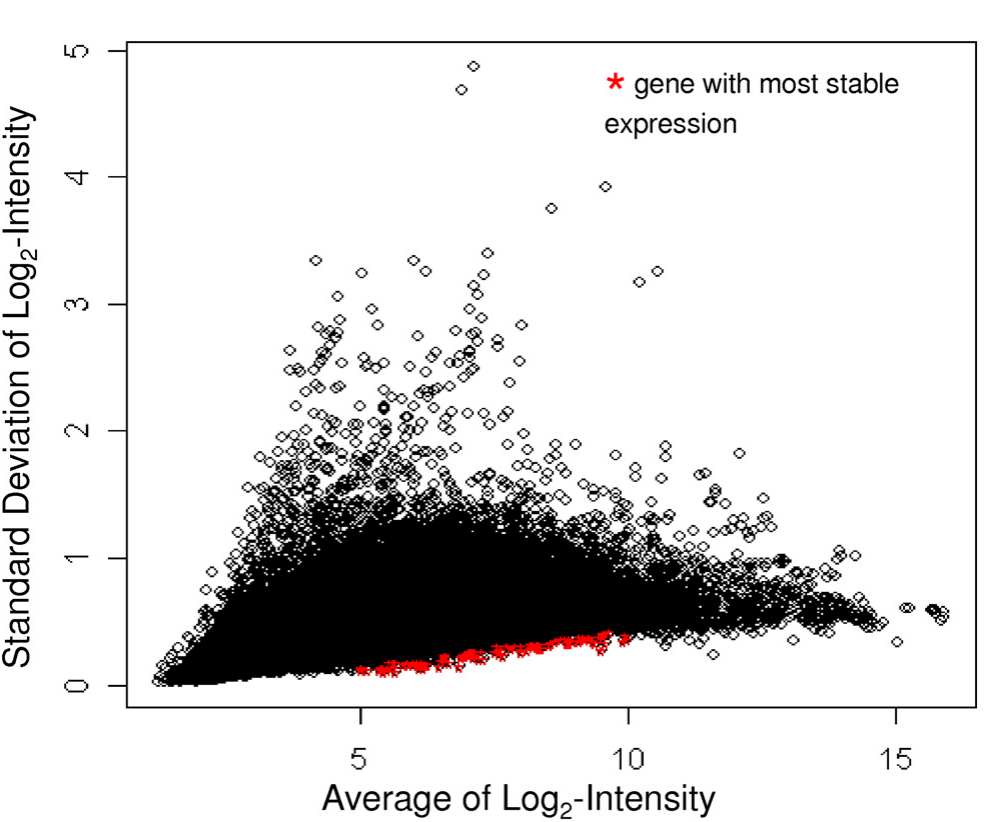
**The Average log_2_- intensity (x-axis) of 39,500 genes from a total of 526 Affymetrix Human 2.0 Plus microarrays is plotted against the standard deviation of the log_2_- intensity (y-axis) of those genes**. Probe-sets highlighted in red are the 100 probe-sets with the lowest standard deviation within an interval of the log_2_-transformed intensity values between 5 and 10.

Our goal was to identify stably expressed genes with a broad range of expression levels from our Affymetrix data on the 526 samples (Table 1). We chose the 20 most stably expressed genes from each of five intensity intervals (n = 100 genes): log_2_-transformed intensity values between 5–6, 6–7, 7–8, 8–9 and 9–10 (Figure 1). For a list of the most stable genes over all of the expression levels, see Additional file 1. Based on our selection criteria, these 100 most stably expressed genes covered a wide range of raw expression values from 32 to 1024. Functional categorization using the Database for Annotation and Visualization, and Integrated Discovery (DAVID, ) showed that 50% of the annotated genes are involved in primary metabolic processes. For the list of the 100 most stably expressed genes on the 526 microarrays see Additional file 1.

### Selection of Candidate Control Genes for Validation by qRT-PCR

**Figure 2:**
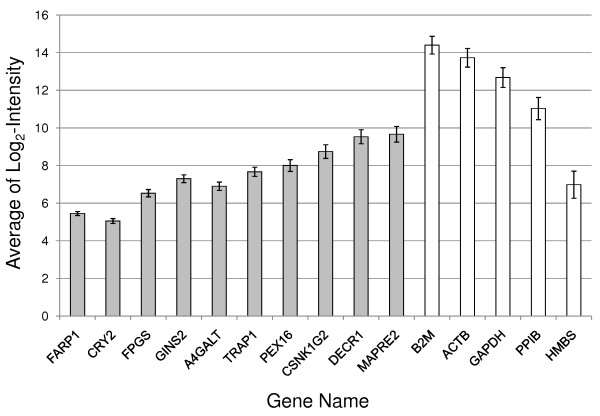
**Average expression calculated from the 526 Affymetrix Human 2.0 Plus microarrays for the candidate control genes selected for qRT-PCR validation**. Average of the log_2_-transformed intensity values ± standard deviation (y-axis) is plotted for each gene. Gray columns represent the candidate control genes selected from Figure 1. White columns represent commonly used house-keeping genes and PPIB.

The set of 100 stably expressed genes on microarrays was filtered down to probe-sets with available gene annotation, as well as to probe-sets detecting only a single known gene (Affymetrix annotation *_at). Based on these criteria, 23 probe-sets were identified (data not shown). From them, 10 candidate genes were selected for validation with, on average, two genes per intensity interval. These 10 candidate genes included TRAP1, DECR1, FPGS, FARP1, MAPRE2, PEX16, GINS2, CRY2, CSNK1G2 and A4GALT. In the validation, we also included four commonly employed reference genes, ACTB, GAPDH, B2M and HMBS. In addition, PPIB was included because it was previously reported to be stably expressed in blood [3]. Each gene and ABI assay information is presented in Table 2. The average expression values from the Affymetrix array data of the genes selected for validation by qRT-PCR are shown in Figure 2. The range of the SDs of the the log_2_-transformed intensity values on the microarrays was between 0.10 and 0.42 for the 10 candidate reference genes on the microarrays (solid bars, Figure 2). The SDs for the remainder genes (open bars, B2M, ACTB, GAPDH, PPIB and HMBS) ranged between 0.47 and 0.72 on the microarrays (Figure 2). Based on the Affymetrix array data alone, FARP1 was the most stably expressed. A general trend of increasing SD with increasing intensity was noted for the candidate reference genes (Figure 2).

**Table 2:** The panel of candidate reference genes chosen for validation by qRT-PCR.

**Gene Symbol**	**ABI Assay ID**	**GenBank ID**	**Gene Name**
FARP1	Hs01120587_m1	BF213279	FERM, RhoGEF (ARHGEF) and pleckstrin domain protein 1 (chondrocyte-derived)
CRY2	Hs00901393_m1	AB014558	cryptochrome 2 (photolyase-like)
FPGS	Hs00191956_m1	NM_004957	folylpolyglutamate synthase
A4GALT	Hs00213726_m1	NM_017436	alpha 1,4-galactosyltransferase
TRAP1	Hs00972326_m1	NM_016292	heat shock protein 75
GINS2	Hs00211479_m1	AW205303	DNA replication complex GINS protein PSF2
PEX16	Hs00937316_m1	AA523441	peroxisomal biogenesis factor 16
CSNK1G2	Hs00176258_m1	AL530441	casein kinase 1, gamma 2
DECR1	Hs00154728_m1	NM_001359	2,4-dienoyl CoA reductase 1, mitochondrial
MAPRE2	Hs00183921_m1	NM_014268	microtubule-associated protein, RP/EB family, member 2
PPIB	Hs00168719_m1	NM_000942	peptidylpropyl isomerase B
HMBS	Hs00609297_m1	NM_000190	hydroxymethyl-bilane synthase
ACTB	Hs00242273_m1	NM_001615	beta-actin
B2M	Hs99999907_m1	NM_004048	beta-2-microglobulin
GAPDH	Hs99999905_m1	NM_002046	glyceraldehyde-3-phosphate dehydrogenase

### qRT-PCR Analysis and Expression of Candidate Control Genes

**Figure 3:**
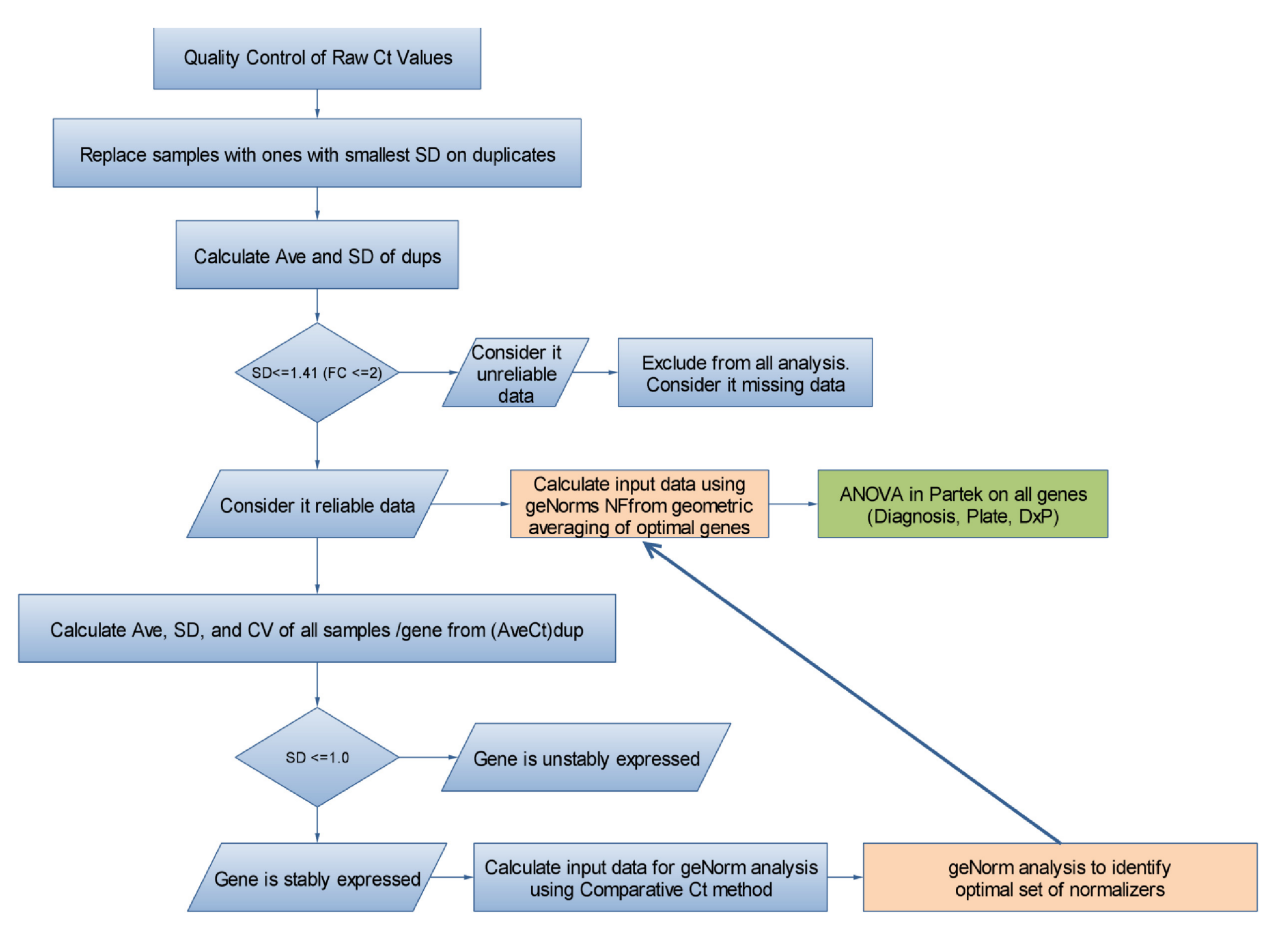
**Flow chart of the data analysis performed for the qRT-PCR studies**.

**Figure 4:**
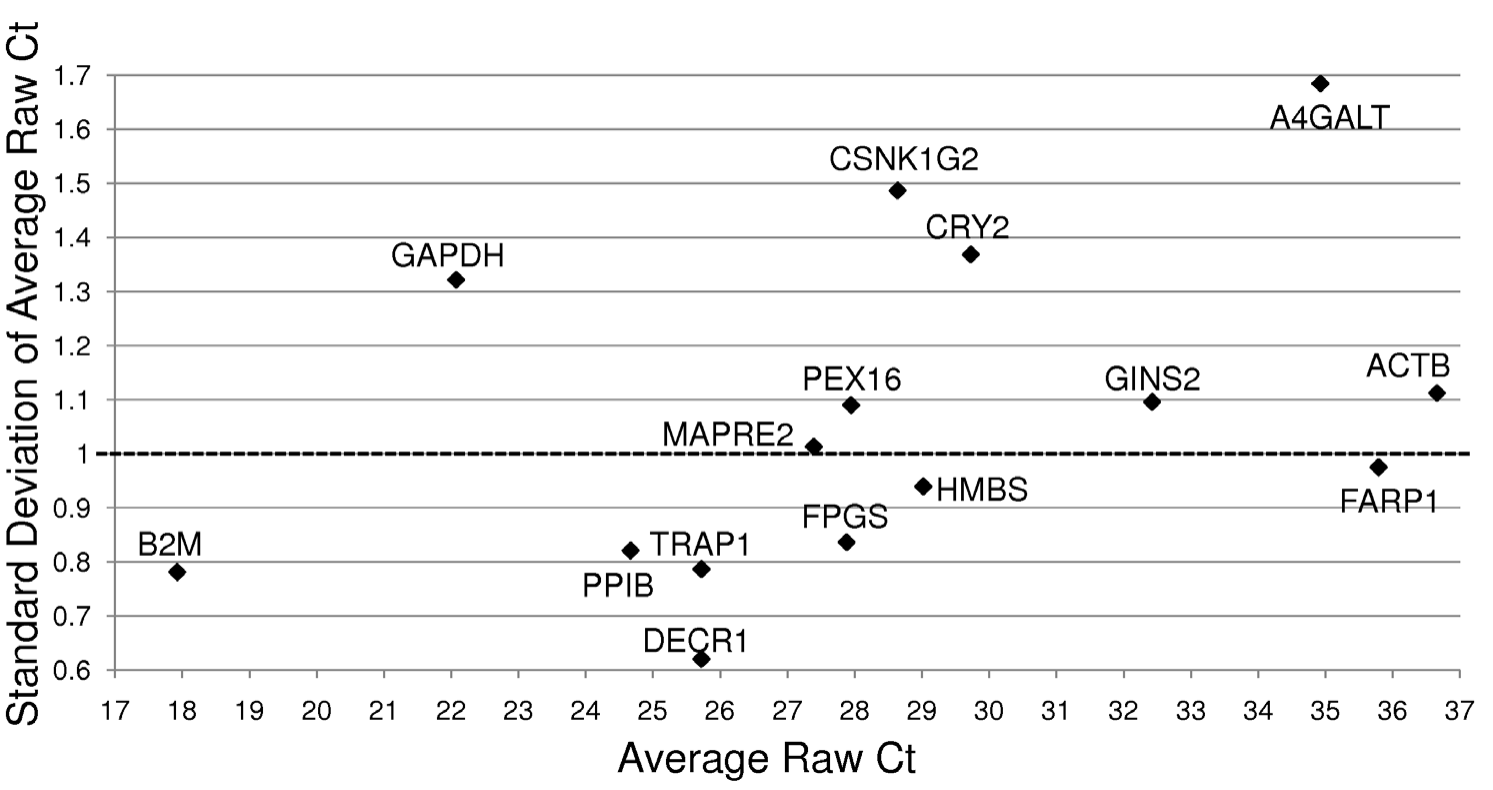
**Expression (average raw Ct, x axis) is plotted versus Standard Deviation of average raw Ct (y axis) for all of the candidate control genes**. qRT-PCR was performed on RNA samples from 20 multiple sclerosis (MS) patients and 20 age and gender matched controls. The average expression and its standard deviation was calculated for each gene for all of the patients combined (n = 40). (Ct = Cycle threshold).

The analysis flowchart presented in Figure 3 describes the steps used to analyze the qRT-PCR data. The subset of 15 candidate reference genes (Table 2), validated on qRT-PCR, exhibited a broad range of expression levels from 17.93 to 36.66 cycle threshold (Ct) values (Table 3, Figure 4). Samples for which the SD on the technical replicates was higher than 1.4 (a fold-change higher than 2) were considered unreliable data and were not included in the downstream analysis, thus reducing the sample size for some assays to lower than 40 (Table 3). Genes that had SD ≤ 1.0 on the average raw Ct values of all samples were considered to be stably expressed. Based on that criteria, TRAP1, B2M, DECR1, PPIB, FPGS, HMBS and FARP1 (Table 3, Figure 4, below the dotted line) showed stable expression over all 40 samples. In contrast, MAPRE2, PEX16, GINS2, ACTB, GAPDH, CRY2, CSNK1G2 and A4GALT did not show stable expression over the samples (Table 3, Figure 4 above the dotted line). The stably expressed genes covered a wide range of expression levels, from a high level of expression of 17.9 Ct for B2M, to low levels of 35.71 Ct for FARP1 (Table 3, Figure 4).

**Table 3:** qRT-PCR on multiple sclerosis patients and normal controls

**Gene Name**	**Ct-avg**	**SD**	**CV**	**n**
TRAP1	25.72	0.62	0.024	40
B2M	17.93	0.78	0.044	40
DECR1	25.72	0.79	0.031	40
PPIB	24.67	0.82	0.033	40
FPGS	27.88	0.84	0.030	40
HMBS	29.02	0.94	0.032	40
FARP1	35.79	0.98	0.027	35
MAPRE2	27.39	1.01	0.037	40
PEX16	27.95	1.09	0.039	40
GINS2	32.42	1.10	0.034	40
ACTB	36.66	1.11	0.030	23
GAPDH	22.07	1.32	0.060	40
CRY2	29.73	1.37	0.046	40
CSNK1G2	28.64	1.49	0.052	40
A4GALT	34.92	1.69	0.048	32

Average raw Cycle threshold values (Ct-avg), standard deviation (SD), coefficient of variability (CV) and sample size (n) for the qRT-PCR analyses of RNA samples from 20 patients with multiple sclerosis compared to 20 age and gender matched control subjects. Because those samples that had a SD > 1.41 for the duplicates were excluded from the analysis, the sample size is less than 40 for some of the genes.

### Expression Stability and Ranking Analysis

To identify the most suitable set of genes for normalization in whole-blood, the average expression stability (M) for those reference genes which showed SD of raw Ct-values ≤ 1.0 on all samples was calculated using the GeNorm tool (see Methods). Those genes included TRAP1, FPGS, B2M, DECR1, PPIB and HMBS (Figure 4). All of these genes also had a SD of the Ct-values for the technical duplicates of ≤ 1.41 and a SD of the raw Ct values of all samples ≤ 1.0. The FARP1 gene was omitted from the analysis of the expression stability because GeNorm does not allow missing values. However, re-analysis where FARP1 was included with the rest of the five stably expressed genes (with n = 35) did not change the final results (data not shown).

An important pre-requisite for estimating the Expression Stability Measure is that the genes are not coordinately regulated. The Gene Ontology terms of the six genes, analyzed in GeNorm, are presented in Table 4. These genes are involved in different biological process, molecular functions and/or are located in different cellular compartments.

**Table 4:** Gene Ontology (GO) Annotation of genes analyzed in GeNorm

**Gene Symbol**	**Gene Name**	**GO Biological Process Term**	**GO Molecular Function Term**	**GO Cellular Component** **Term**
FPGS	folylpolyglutamate synthase	nucleobase, nucleoside, nucleotide and nucleic acid metabolic process glycine metabolic process one-carbon compound metabolic process biosynthetic process nucleoside metabolic process folic acid and derivative biosynthetic process	nucleotide binding Tetrahydrofolylpoly- glutamate-synthase activity ATP binding ligase activity	cytoplasm mitochondrion cytosol

TRAP1	TNF receptor-associated protein 1	protein folding	nucleotide binding receptor activity tumor necrosis factor receptor binding ATP binding unfolded protein binding	Mitochondrion

DECR1	2,4-dienoyl CoA reductase 1, mitochondrial	metabolic process oxidation reduction	catalytic activity Binding 2,4-dienoyl-CoA reductase(NADPH) activity oxidoreductase activity	mitochondrion

PPIB	peptidylprolyl isomerase B (cyclophilin B)	protein folding	peptidyl-prolyl cis-trans isomerase activity protein binding isomerase activity peptide binding unfolded protein binding	endoplasmic reticulum endoplasmic reticulum lumen melanosome

HMBS	hydroxymethyl-bilane synthase	porphyrin biosynthetic process heme biosynthetic process tetrapyrrole biosynthetic process	hydroxymethylbilane synthase activity transferase activity	cytoplasm

B2M	beta-2-microglobulin	antigen processing and presentation of peptide antigen via MHC class I immune response	protein binding	Golgi membrane extracellular region plasma membrane early endosome membrane MHC class I protein complex

**Figure 5:**
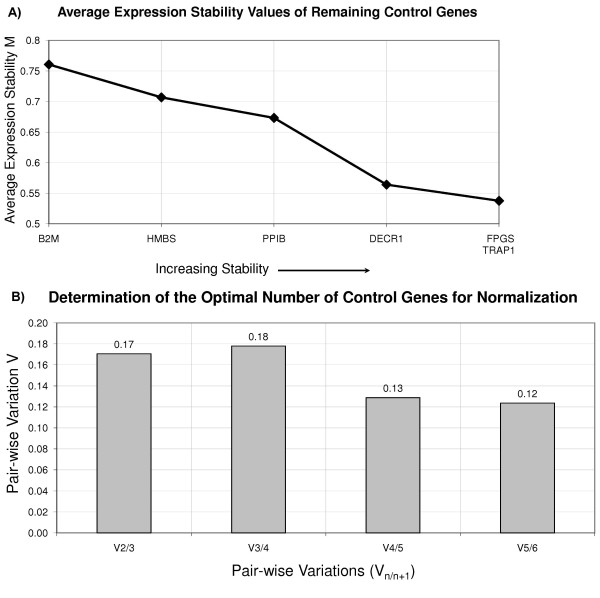
**Identification of the most stably expressed reference genes using GeNorm analysis**. RNA samples from whole-blood from 20 MS patients and 20 matched controls were analyzed using qRT-PCR. A. Average expression stability values (M) of the reference genes during step-wise exclusion of the least stable control gene. The x- axis shows the gene with the highest M value (the lowest stability) for this set of genes. The least stable gene was excluded after each iteration. B. Determination ofthe optimal number of reference genes for normalization. V is the pair-wise variation of two sequential normalization factors. The least number of genes for each V<0.15 was selected as the optimal set of genes for normalization [6]. In this study, the four most stably expressed genes (TRAP1, FPGS, DECR1 and PPIB) represent the optimal set of genes for normalization.

The GeNorm algorithm calculates the M value of a gene based on the average pair-wise variation between all genes in the analysis. Figure 5A plots the average expression stability of the six reference genes. This curve was generated by a step-wise exclusion of the least stable reference gene. It shows that B2M was the least stable, while TRAP1 and FPGS were the two most stably expressed genes in whole blood of patients with MS and gender and age matched controls.

Determination of the optimal number of reference genes for normalization of the dataset uses a metric called V. It is the pair-wise variation of two sequential normalization factors. For consistency with the recommendations of Vandesompele et al [6], the least number of genes for which V<0.15 should be selected as the optimal set of genes for normalization. In the study, the four most stably expressed genes, TRAP1, FPGS, DECR1 and PPIB, represent the optimal set of genes for normalization (Figure 5B). We estimated the gene expression stability and ranking of the control genes using an alternative algorithm (NormFinder). The relative ranking of the genes was essentially the same as the one obtained using GeNorm. The most stable gene was TRAP1 (stability value 0.066), followed by FPGS (0.067), DECR1 (0.078), PPIB (0.079), HMBS (0.095) and B2M (0.108). The main differences between the GeNorm algorithm (reference) and the model-based variance estimation approach in NormFinder [27] is that the latter takes into account the inter- and intra-group variation and uses direct estimation of variances as opposed to pair-wise variation.

### Analysis of Variance (ANOVA)

To identify genes that showed differential expression between the MS subjects and the healthy controls, an ANOVA was performed on the qRT-PCR data. Diagnosis and each PCR-plate were included as factors. CSNK1G2 showed significant differences in expression between MS and control samples (Table 5, p < 0.05). However, if multiple-comparison correction is applied to this p-value, it is not statistically significant. Expression of the remaining genes did not differ significantly between the matched pairs (Table 5). No significant p-values (p ≤ 0.05) for plate, nor for the Diagnosis-versus-plate interaction were found, suggesting no significant technical bias in the study (data not shown). The fold-change of expression between the MS and the healthy controls for the stably expressed genes (Figure 4, below the dotted line) was closest to 1.0 for FPGS (Table 5). ACTB and A4GALT fold-changes could not be estimated in Partek due to multiple missing values. The greatest deviation from the 1.0 ratio for all the genes was observed for CSNK1G2. We also used the ΔΔCt method [28], as described in the Methods section, to calculate fold-change. B2M showed the smallest deviation from the 1.0 ratio for the most stably expressed genes (Table 5). TRAP1, identified by GeNorm as one of the most stably expressed genes, was used as the Normalizer; therefore, its ratio is not relevant. The highest deviation from the 1.0 ratio for all the genes was observed for CSNK1G2, as expected.

**Table 5:** Diagnosis effect on the candidate control genes.

	**ΔΔ**Ct Method	Comparative Ct Method	
	
	Normalized to TRAP1	Normalized to Optimal Set of 4 Genes*	
	
Gene	Fold Change (MS/H)	Fold Change (MS/H)	p-value(Diagnosis)
FPGS	1.08	1.00	0.964
ACTb	1.08	ND	0.788
GINS2	1.21	1.11	0.691
HMBS	1.12	1.06	0.581
B2M	-1.04	-1.10	0.575
FARP1	1.34	1.16	0.559
A4GALT	-1.01	ND	0.416
TRAP1	**	-1.08	0.342
GAPDH	-1.45	-1.61	0.198
DECR1	-1.09	-1.11	0.161
PPIB	1.32	1.22	0.155
PEX16	1.32	1.23	0.073
MAPRE2	1.42	1.30	0.072
CRY2	1.58	1.45	0.065
CSNK1G2	1.76	1.63	0.026

Expression of the candidate reference genes in whole blood of multiple sclerosis (MS) subjects is compared to age and gender matched control subjects using the **ΔΔ**Ct Method and the Comparative Ct Method. Genes are arranged in the order of decreasing p-value for diagnosis.

*ANOVA: μ + Diagnosis + Plate + (Diagnosis × Plate) + ε

**TRAP1 fold change not calculated since that gene was used as a normalizer.

**Figure 6:**
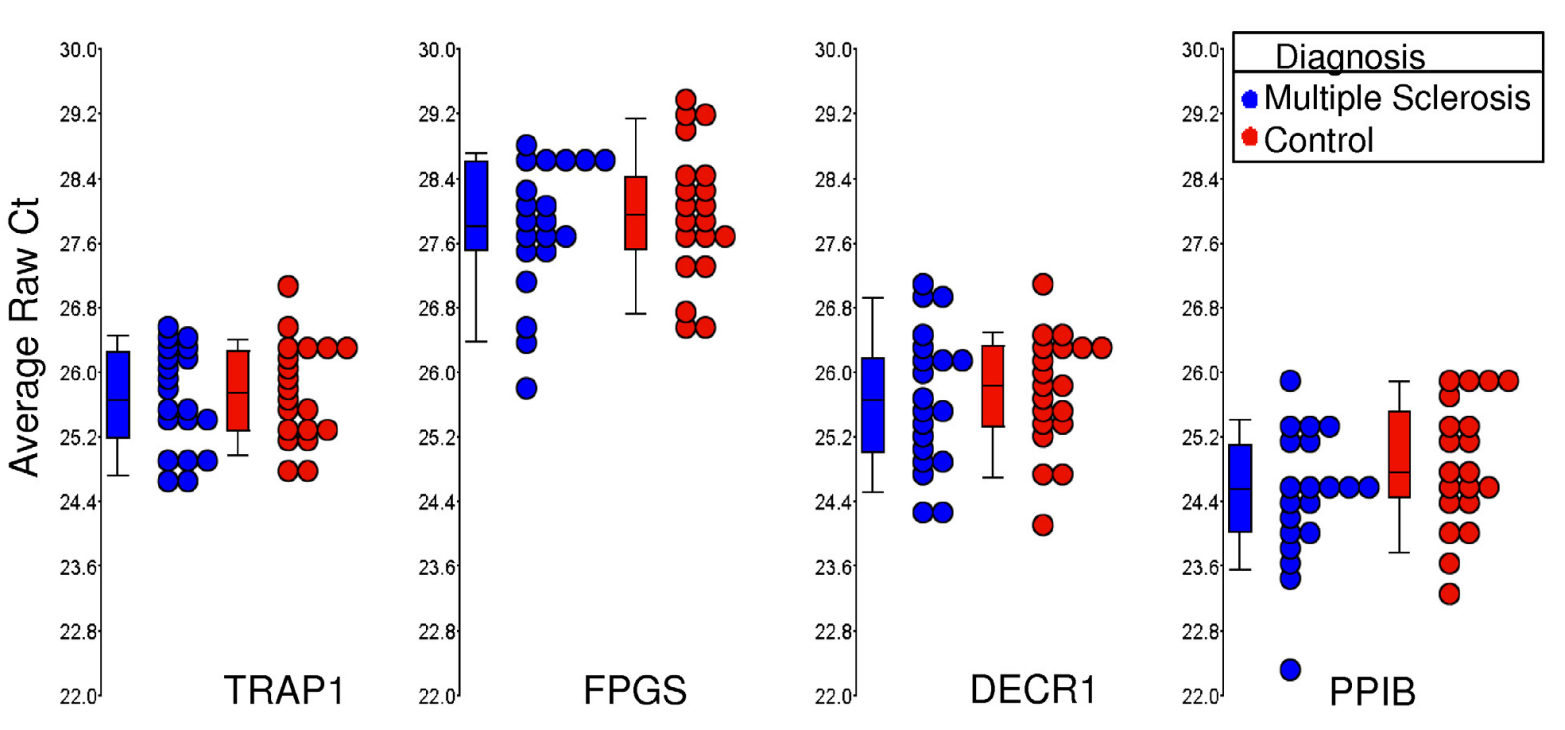
**RNA expression values are shown for the four most stably expressed genes (TRAP1, FPGS, DECR1 and PPIB) for multiple sclerosis (MS) and controls subjects**. Circles represent individual qRT-PCR Ct average raw values for MS samples (blue, n = 20) and age and gender matched controls (red, n = 20). Boxes represent the 25^th ^and 75^th ^quartile and lines represent the median. Whiskers represent the range of data for the 10^th ^and 90^th ^quartile.

Data on the four genes, which constitute the optimal set of normalizers (TRAP1, FPGS, DECR1 and PPIB) on the MS and healthy control samples are shown separately as box-and-whisker plots (Figure 6). Data for both the ΔΔCt and Comparative Ct methods for MS versus control patients are shown in Table 5, with similar results being obtained with both methods.

## Discussion

Gene expression techniques such as qRT-PCR, microarrays and Northern blotting require accurate normalization methods in order to obtain reliable, quantitative data. A common approach is to use an endogenous reference gene. The purpose of the reference gene(s) is to remove differences not attributable to real biological variation. However, numerous reports indicate that differences in the expression levels of commonly used endogenous reference genes vary considerably between different tissues and between different experimental conditions. Thus, there are no universal reference genes for all tissues or experimental conditions [2,3,29-35].

To address this problem, the best reference genes must be determined for each individual tissue or experimental setting. Moreover, a combination of several endogenous control genes produces more reliable normalization than any single control gene [6]. We therefore examined the expression of over 39,000 genes in 526 samples of whole blood from men, women and children that included healthy controls and individuals with a variety of different diseases. The 100 most stably expressed probe-sets were identified based upon having the least variance across all of these samples over a broad range of expression values. These 100 genes could also be useful as endogenous internal controls for microarray studies – an approach not commonly used at present for microarray studies. Fifty percent of the annotated genes were involved in primary metabolic processes. Thus these genes appear to be good ''housekeeping genes'' for human blood because of their stable expression across various ages, genders, and diseases.

qRT-PCR validation was then performed on 10 candidate reference genes, four commonly used reference genes (ACTB, GAPDH, B2M, and HMBS) and PPIB, which is reported to be stably expressed in blood [3]. The two frequently used control reference genes in nervous tissue, ACTB and GAPDH, were not stably expressed under our experimental conditions and are therefore not suitable reference genes for human whole blood. They have also been shown to vary in a variety of experimental settings and tissues [2,33,36-39], including in whole blood [3]. B2M and PPIB showed stable expression in our study. They were not identified as stably expressed in our Affymetrix data because their expression was higher than our upper limit for selection. PPIB was also reported to be stably expressed in whole blood of patients with local and systemic inflammatory syndromes and healthy controls [3]. HMBS had a high SD based on our Affymetrix arrays on the 526 samples (SD of log-2-transformed values = 0.72). For comparison, the most stably expressed genes in the same intensity interval had a standard deviation (SD) of the log-2-transformed values around 0.2. HMBS, however, showed stable expression when using qRT-PCR in the MS versus healthy controls experiment. Closer inspection of the expression stability on the 526 samples from the Affymetrix chips showed that the HMBS SD of the log-2-transformed values for most of the studies was around 0.5, but it was 0.94 for the stroke study. This result suggests that there were study-specific differences for stroke versus the other diseases, leading to its unstable expression. Our conclusion that HMBS was unstably expressed across our 526 samples is consistent with a previous study reporting that HMBS was unstably expressed in leukocytes [6]. Among the 10 candidate reference genes, TRAP1, DECR1, FPGS, and FARP1 showed the most stable expression.

In most research studies, the target genes or genes regulated within the study are expressed at different levels. In such situations, it is preferable if the comparable endogenous or "housekeeping genes" are expressed at comparable levels as the target gene. This is essential if the microarray, RT-PCR or Northern blotting platform is non-linear at very low or at very high levels. Thus, having a set of endogenous reference genes that are stably expressed at different levels makes it possible to help select those whose expression is comparable to the target genes.

An optimal set of reference genes was identified based on the expression stability measure M as utilized in the GeNorm algorithm. It is based on the concept that the ratio between two ideal reference genes should always be constant regardless of the experimental conditions used. An important assumption here is that the genes in the analysis are not coordinately regulated and have independent functions. The six genes analyzed in GeNorm, seem to be involved in different biological processes, molecular functions and/or are located in different cellular compartments (Table 4). TRAP1 and PPIB are involved in the same biological process however their cellular compartmentalization is different. TRAP1 is a mitochondrial molecular chaperone also known as heat shock protein 75 (Hsp75). PPIB, encoding the cyclophillin B protein, is a cytoplasmic peptide isomerase. The broad spectrum of functions of the stably expressed genes makes it less likely that they are coordinately regulated.

An advantage of the approach taken in this study is that we performed a whole-genome survey of a large number of subjects of different ages, genders and diseases to identify the most stably expressed genes in whole blood. Drawbacks to this approach include the fact that it is an array-based discovery method that is less likely to reliably detect very low expressing genes. In addition, the subjects chosen do reflect some bias related to the diseases studied, and reflect other biases in the subject selection – such as predominantly hospitalied and male patients.

It should be emphasized that the ultimate usefulness of the proposed endogenous reference genes for whole blood has not been demonstrated in this study. To do this, future studies must show that after correcting technical and individual variability according to the control genes, the discriminatory power of the diagnostic genes should be substantially improved.

It is important to point out the utility of the reference genes identified here for use in clinical human testing. Should the reference genes identified in this study provide sufficiently accurate normalization, then genes of interest could be normalized to these reference genes in healthy individuals and in individuals with a given disease or physiological condition. Once reference expression levels and standard deviations are derived in a large group of healthy individuals, deviations could be identified in target individuals using these endogenous genes for normalization without the need for repeated samples from normal, healthy individuals. For non-clinical research studies, such normalization to the same endogenous reference genes would allow for comparison across studies.

## Conclusion

We took advantage of the large amount of expression data on 33,000 genes from 526 individuals to select genes that had stable expression in whole blood. This gave us an added advantage to assess not only the gene expression of commonly used housekeeping genes but to perform a whole genome survey for stably expressed genes over multiple ages, disease conditions and different expression levels. Ranking based on the expression stability from qRT-PCR data of the candidate control genes and estimation of the minimum number of genes needed for nomalization showed that the fewest, most stably expressed genes needed for acurate normalization in RNA expression studies of human whole blood is a combination of TRAP1, FPGS, DECR1 and PPIB. These genes should be useful as normalization genes for a wide-range of whole blood expression studies in humans.

## Competing interests

The authors declare that they have no competing interests.

## Authors' contributions

BSS, MA, WLW and FRS designed the study, analyzed the results and wrote the manucript. MA provided the samples from the MS patients for the RT-PCR and array studies. YT, HX, PA, XA, DL, BPA, IHL, JPG, RJT, GCJ and LL either helped collect samples, helped analyze the data from the microarray studies and/or read and proof read the manuscript. JPG supervised the processing of a number of the arrays used in these studies. All authors read and approved the final manuscript.

## Pre-publication history

The pre-publication history for this paper can be accessed here:



## Supplementary Material

Additional file 1 **100 Most Stable Genes and 10 Affy Candidate Reference Genes**. Information on sheet 2 represents the 100 most stable genes including Affynetrix probe-set IDs, reference public IDs, gene symbols, and expression levels. The data on sheet 2 represents the 10 Affymetrix candidate reference genes with probe-sets, ABI assay, and expression levels information. Click here for file
